# Early pandemic use of face masks in Papua New Guinea under a mask mandate

**DOI:** 10.5365/wpsar.2023.14.1.998

**Published:** 2023-03-28

**Authors:** Mark Raphael, Angela Kelly-Hanku, David Heslop, Danielle Hutchinson, Mohana Kunasekaran, Ashley Quigley, Raina MacIntyre

**Affiliations:** a Papua New Guinea Institute of Medical Research, Goroka, Eastern Highland Province, Papua New Guinea.; b Public Health Intervention Research Program, Kirby Institute, University of New South Wales, Sydney, New South Wales, Australia.; c School of Public Health and Community Medicine, University of New South Wales, Sydney, New South Wales, Australia.; d Biosecurity Program, Kirby Institute, University of New South Wales, Sydney, New South Wales, Australia.; e School of Public Affairs, College of Public Service and Community Solutions, Arizona State University, AZ, United States of America.

## Abstract

**Objective:**

During the coronavirus disease (COVID-19) pandemic, face mask wearing was mandated in Port Moresby, Papua New Guinea in July 2020, but compliance was observed to be low. We aimed to determine the frequency of face mask wearing by the general public in Papua New Guinea under the mask mandate.

**Methods:**

To estimate compliance with the mandate, we analysed photographs of people gathering in Port Moresby published between 29 September and 29 October 2020. Photo-epidemiology was performed on the 40 photographs that met pre-defined selection criteria for inclusion in our study.

**Results:**

Among the total of 445 fully visible photographed faces, 53 (11.9%) were observed wearing a face mask over mouth and nose. Complete non-compliance (no faces wearing masks) was observed in 19 (4.3%) photographs. Physical distancing was observed in 10% of the 40 photographs. Mask compliance in indoor settings (16.4%) was higher than that observed in outdoor settings (9.8%), and this difference was statistically significant (*P* < 0.05). Mask compliance was observed in 8.9% of large-sized gatherings (> 30 people), 12.7% of medium-sized gatherings (11–30 people) and 25.0% of small-sized gatherings (4–10 people; photographs with < 4 people were excluded from analysis).

**Discussion:**

We found very low population compliance with face mask mandates in Papua New Guinea during the pre-vaccine pandemic period. Individuals without face coverings and non-compliant with physical distancing guidelines are considered to be in a high-risk category for COVID-19 transmission particularly in medium- and large-sized gatherings. A new strategy to enforce public health mandates is required and should be clearly promoted to the public.

The coronavirus disease (COVID-19) pandemic has affected all countries but has had a disproportionate impact on low- and middle-income countries such as Papua New Guinea. (*1*) Papua New Guinea is one of the world’s most diverse countries – geographically, ethnically, linguistically, environmentally and culturally. The majority of the population (> 85%) lives in rural villages, which are often difficult to access due to the country’s challenging terrain. (*2*) As of 3 February 2023, there were 46 750 confirmed cases of COVID-19 and 670 confirmed deaths in Papua New Guinea. (*3*) However, these case numbers are likely underestimates due to low testing rates. (*4*)

Several factors have increased the vulnerability of the population of Papua New Guinea to COVID-19. Cultural practices and events unique to Papua New Guinea, including funeral practices (*haus krai*), religious and sporting gatherings, as well as cultural events such as singings, have the potential to cause widespread transmission of severe acute respiratory syndrome coronavirus 2 (SARS-CoV-2) within communities. Traditional greeting practices, which include shaking hands or embracing, (*5*) and crowded community living also pose a high risk for transmission of SARS-CoV-2 and other diseases, such as tuberculosis. (*6*) In Papua New Guinea, the average household size is 6.24 people in rural areas and 8.01 in urban areas. The highest density is in the National Capital District (NCD), with 9.19 people per household. (*7*) These factors, combined with poor sanitation and hygiene practices, greatly increase the risk of SARS-CoV-2 transmission in many communities. (*8*) As the transmission of SARS-CoV-2 is predominantly via an airborne route, crowded gatherings have been identified as an important contributor to the spread of COVID-19. Although large mass gatherings have been frequently cited as a major source of case transmission, the so-called super-spreader events, gatherings of less than 100 people in private or enclosed public places, have been shown to cause the highest incidence of new cases, suggesting that density and ventilation may have more effect on transmission risk than crowd size. (*9*)

In 2020, as COVID-19 case numbers increased globally, Papua New Guinea adopted several public health and social measures to prevent community transmission, including travel restrictions, quarantine and isolation measures, physical distancing and face mask wearing. (*10*) However, despite the government-imposed restrictions, daily routine mobility persisted, especially in rural areas or communities where enforcement of restrictions was limited. (*11*) On 3 October 2020, following a plateau in case numbers across the nation, most of the measures were relaxed, including restrictions on domestic and international flights, and business premises and recreational centres reopened. (*3*) In contrast, the face mask mandate, implemented on 23 July 2020, remained in place in areas with continued levels of community transmission such as Port Moresby, NCD, which had accounted for 70 of the 91 cases nationwide from the month of September 2020. (*3*, *12*) As of 4 October 2020, NCD accounted for 60% (*n* = 322) of the country’s cumulative reported cases. (*3*)

Evidence suggests that mask wearing by healthy people in community settings provides protection against SARS-CoV-2 infection, (*13*) and face mask wearing is also a well established method of source control. (*14*) In addition, a study conducted in Australia, the United Kingdom of Great Britain and Northern Ireland and the United States of America showed that mandating the use of face masks results in higher usage of masks. (*15*) In Papua New Guinea, however, anecdotal evidence was suggestive of widespread non-compliance with mandatory face mask wearing. (*16*) This study therefore aimed to estimate the frequency of face mask wearing by the general public in Port Moresby during the early stages of the COVID-19 pandemic before vaccines being available, when community transmission was established and mask wearing was mandated.

## Methods

A search was conducted in the online archives of *The National* newspaper for photographs of gatherings in Port Moresby between 29 September and 29 October 2020. This month-long time period was chosen because at that time the mask mandate remained in place in NCD due to ongoing community transmission (rather than a COVID-19 surge), while across the country all other restrictions had been lifted, including those on schools, sporting matches and international travel. (*12*) A list of sources for each photograph is given in **Supplementary Table 1**. Only one newspaper was searched to avoid duplication of photographs capturing the same event.

Each photograph was assessed against a set of inclusion and exclusion criteria (**Table 1**). To be included in the study, photographs had to be of gatherings held in Port Moresby between 29 September and  29 October 2020, spontaneous and of sufficient quality with clear visibility for easy counting purposes. In addition, there had to be a minimum of four people in the photograph.

**Table 1:** Inclusion and exclusion criteria for photograph selection

Inclusion criteria	Exclusion criteria
Photograph captured in Port Moresby between 29 September and 29 October 2020	Photograph taken outside of the reporting period
Photograph clearly visible for purpose of counting	Photograph is blurred or unclear
Photograph used only once	Duplicate photograph
A minimum of four people in the photograph	Less than four people in the photograph
Photograph is taken spontaneously/unplanned	Photograph is arranged, planned or orchestrated (e.g. group portrait)

### Data analysis

Photo-epidemiology was performed by two reviewers independently (DH and MK); any discrepancies were resolved by a third reviewer (AQ). (*17*, *18*) A manual head count was done to determine the gathering size, and a count of all faces with the mouth and nose visible was performed. Finally, a mask count was performed; to be included in the mask count, masks had to cover both the mouth and nose (masks covering only the mouth were not included in the count). Masks could be of the surgical, N95, disposable or cloth types; any other form of face covering was excluded from the count. Mask wearing was calculated as the number of people wearing a mask as a percentage of the number of visible faces. Each photograph was counted twice by each reviewer, with the average of the four counts used for analysis. Inter-rater reliability score was calculated to ascertain the level of agreement between the two reviewers (DH and MK) who performed the counting of the photographs. The statistical package R (version 3.6.3) was used for this analysis, with the Kappa coefficient obtained using the “psych” and “irr” packages. (*19*) Inter-rater reliability was high: Cicchetti-Allison weighted Kappa = 0.995 (confidence interval: 0.991–0.997), (*20*) implying an almost perfect level of agreement between the two reviewers.

The United States Centers for Disease Control and Prevention defines small gatherings as informal and usually occurring with family and friends within a regular social gathering, and large gatherings as consisting of many people from multiple households in a public space, such as conferences, sporting events, festivals and large parties. (*21*) As risk of COVID-19 transmission is considered to vary according to the size of gatherings, (*22*) photographs were further categorized according to small (4–10), medium (11–30) and large (> 30) in-person gatherings, which was extrapolated from the head count (as a minimum number of people at the gathering). (*22*)

The photographs were also examined for evidence of physical distancing; photographs were rated as “yes” if people were more than 1.5 m apart in the photographs, and “no” if the distance between people was less than this. Photographed gatherings were also categorized as either “indoor” or “outdoor.” We calculated the proportions of mask compliance by setting (indoor vs outdoor), presence of physical distancing (yes vs no) and gathering size (small vs medium vs large). To ascertain whether there were significant differences in mask wearing compliance by setting and presence of physical distancing, a two-sample z-test was used. To assess the effect of gathering size on compliance, we used a χ^2^ test (3x2 contingency table). A 95% level of significance was used for all statistical tests.

## Results

A total of 72 photographs published from 29 September to 29 October 2020 were identified by the search and screened for eligibility; 31 photographs were subsequently excluded because they had the appearance of being “non-spontaneous” (i.e. staged), and one was excluded because it was of fewer than four people. Analysis was performed on the 40 remaining photographs that met all study inclusion criteria. A total of 944 people were captured in the photographs; 445 faces were sufficiently visible to assess mask wearing. Averaged across all 40 photographs, the proportion of people observed wearing a mask was 11.9% (*n* = 53) (**Table 2**).

**Table 2:** Mask compliance according to setting, presence of physical distancing and gathering size

Variable	Number of photographs (% of column total)	Total number of faces (% of column total)	Mask compliance	
			Number of faces with masks (% of faces)	*P*
**Setting**				
Indoor	11 (27.5%)	140 (31.5%)	23 (16.4%)	< 0.05
Outdoor	29 (72.5%)	305 (68.5%)	30 (9.8%)	
Total	40 (100.0%)	445 (100.0%)	53 (11.9%)	
**Physical distancing present**				
Yes	4 (10.0%)	37 (8.3%)	14 (37.8%)	< 0.0001
No	36 (90.0%)	408 (91.7%)	39 (9.6%)	
Total	40 (100.0%)	445 (100.0%)	53 (11.9%)	
**Gathering size**				
Small (1–10 people)	8 (20.0%)	32 (7.2%)	8 (25.0%)	< 0.0001
Medium (11–30 people)	23 (57.5%)	221 (49.7%)	28 (12.7%)	
Large (> 30 people)	9 (22.5%)	192 (43.1%)	17 (8.9%)	
Total	40 (100.0%)	445 (100.0%)	53 (11.9%)	

Note: Final count numbers are expressed as an average of three reviewers and represented as whole persons.

In nearly half of the photographs (*n* = 19; 47.5%), no one was wearing a mask. Of these 19 photographs where zero mask compliance was observed, 4 were of indoor settings and 15 were of outdoor settings; 18 of the 19 exhibited no evidence of physical distancing. Nearly two thirds of the 19 photographs showing no mask compliance (*n* = 12; 63.1%) were of small-sized gatherings; 5 (26.4%) were of medium-sized gatherings and 2 (10.5%) were of large-sized gatherings (*P* < 0.05).

**Table 2** shows the levels of mask compliance by setting (indoor vs outdoor), presence of physical distancing (yes vs no) and gathering size (small vs medium vs large) across all 40 photographs. There were statistically significant differences between the proportion of people wearing masks by setting, presence of physical distancing and gathering size. The proportion of faces with masks was higher in indoor settings than in outdoor settings (16.4% vs 9.8%; *P* < 0.05). The prevalence of mask wearing was higher among those who were observed practising physical distancing relative to those who were not (37.8% vs 9.6%; *P* < 0.0001). Finally, mask compliance was highest among those attending small gatherings (25.0%), followed by those participating in medium-sized gatherings (12.7%). At 8.9%, the lowest level of compliance was observed among those attending large gatherings.

## Discussion

Using photo-epidemiology, we found low levels of compliance with government-mandated regulations relating to face mask wearing in Port Moresby during October 2020. An overall compliance of just 11.9% was observed; compliance was especially low among people attending outdoor events and medium- and large-sized gatherings, highlighting the potential for higher COVID-19 transmission in these settings.

In Papua New Guinea, sociocultural norms, as well as personal, social and environmental barriers, are likely to impact population attitudes and compliance with public health measures. (*23*) In July 2020, Papua New Guinea formally adopted *Niupela Pasin* – guidelines for the “new normal” in the time of the COVID-19 pandemic. These guidelines included physical distancing, hand hygiene, respiratory etiquette and the use of face masks when physical distancing is not possible. (*24*) However, widespread adoption of *Niupela Pasin*, including hand washing and face mask wearing, failed to materialize and there was little evidence of compliance. (*25*) Cultural factors and social barriers, such as a fear of being considered sick with COVID-19 when wearing a mask, being stigmatized when wearing a mask or fear of judgement when wearing a mask, have been suggested as possible reasons for low compliance.

Mask wearing has a strong cultural significance in Papua New Guinean societies and features prominently in many traditional ceremonies and festivals. Mask types differ by region and serve a variety of purposes, including representation of totems, entertainment, intimidation and concealment of the wearers’ identity. In the majority of circumstances, traditional masks are worn by men. (*26*) Wearing of face masks for protection from an airborne virus was an unfamiliar context, with high potential for cultural resistance and low uptake.

In a study of risk perceptions and responses to COVID-19 conducted early in the pandemic on a university campus, both students and lecturers reported physical distancing as being contrary to Papua New Guinean culture. Hugging, shaking hands and standing closely in groups are seen as cultural practices and “a way of life” that is very difficult to stop. (*27*) This study also noted that although there were directions by university management to wear a mask on campus, compliance diminished quickly, and there was a lack of compliance with mask use on campus by both students and lecturers. (*27*)

Other possible reasons for low compliance include difficulties in obtaining face masks because of a lack of supply, a lack of accessibility and/or high cost. Supplies of personal protective equipment (PPE) were indeed limited in Papua New Guinea during the height of the COVID-19 pandemic, with most PPE donated by foreign organizations for use within the health service. (*28*) Many people could not afford disposable masks for daily use or had difficulty cleaning reusable masks due to lack of water supply. Strategies aimed at removing those barriers would be needed to improve face mask use.

Understanding the reasons behind the lack of enforcement of the mask mandate would also help to strengthen COVID-19 prevention efforts. Although the Papua New Guinea National Pandemic Act 2020 specifies which geographical areas and settings require mandatory face mask wearing, when people should be exempted and who is responsible for enforcing the orders, it does not describe how the mandate should be enforced. (*29*) Similar regulatory gaps were evident in other countries, such as the United States of America, where the state governments relied on businesses to enforce the mandate, but businesses expected the government to enforce the mandate. (*30*) This implies that any attempts to improve face mask wearing through mandatory regulations would need to be accompanied by a greater level of community engagement and better health promotion messaging.

This study is subject to some limitations. Photo-epidemiology is not a well established science. However, in the absence of more rigorous methods for monitoring mask wearing, it serves as an appropriate surrogate measure and has been used effectively in other fields of research. (*17*, *18*) The sample we captured may not be representative of the whole community. Physical distancing calculations were estimated and may have introduced measurement error. Counting issues may arise as the visibility of faces in photographs is subjective and could differ between viewers. We addressed this by calculating an inter-rater reliability score to assess variability between reviewers. We acknowledge that some people may have taken their mask off for a photo but took this into account by excluding posed photographs. A qualitative study to assess behaviour around taking photographs while wearing a face mask would have addressed this issue but was beyond the scope of this study. The photographs taken during the study period may not be representative of normal daily life. Finally, the level of community transmission declined during the study period, with cases decreasing in NCD from 70 in the previous month (September 2020) to 22 confirmed cases during the study period (October 2020), (*3*, *4*, *12*) which may have impacted attitudes towards mask wearing.

In conclusion, we found very low face mask compliance in Port Moresby, Papua New Guinea during a mask mandate in the period before vaccines being available. Health authorities in Papua New Guinea will require better strategies to address the individual, social and cultural barriers to improve population attitudes towards face mask use and prevent SARS-CoV-2 transmission, especially in high-risk gatherings.
